# Functional modulation and directed assembly of an enzyme through designed non-natural post-translation modification†
†Electronic supplementary information (ESI) available: Detailed experimental methods, supplementary Fig. 1 to 11 and supplementary Tables 1 to 3. See DOI: 10.1039/c4sc03900a



**DOI:** 10.1039/c4sc03900a

**Published:** 2015-03-31

**Authors:** Andrew M. Hartley, Athraa J. Zaki, Adam R. McGarrity, Cecile Robert-Ansart, Andriy V. Moskalenko, Gareth F. Jones, Monica F. Craciun, Saverio Russo, Martin Elliott, J. Emyr Macdonald, D. Dafydd Jones

**Affiliations:** a School of Biosciences , Cardiff University , Cardiff , UK . Email: jonesdd@cardiff.ac.uk; b School of Physics and Astronomy , Cardiff University , Cardiff , Wales , UK; c Centre for Graphene Science , University of Exeter , Exeter , Devon , UK

## Abstract

Post-translational modification (PTM) modulates and supplements protein functionality. In nature this high precision event requires specific motifs and/or associated modification machinery. To overcome the inherent complexity that hinders PTM's wider use, we have utilized a non-native biocompatible Click chemistry approach to site-specifically modify TEM β-lactamase that adds new functionality. *In silico* modelling was used to design TEM β-lactamase variants with the non-natural amino acid *p*-azido-l-phenylalanine (azF) placed at functionally strategic positions permitting residue-specific modification with alkyne adducts by exploiting strain-promoted azide–alkyne cycloaddition. Three designs were implemented so that the modification would: (i) inhibit TEM activity (Y105azF); (ii) restore activity compromised by the initial mutation (P174azF); (iii) facilitate assembly on pristine graphene (W165azF). A dibenzylcyclooctyne (DBCO) with amine functionality was enough to modulate enzymatic activity. Modification of TEM^W165azF^ with a DBCO–pyrene adduct had little effect on activity despite the modification site being close to a key catalytic residue but allowed directed assembly of the enzyme on graphene, potentially facilitating the construction of protein-gated carbon transistor systems.

## Abstract 2

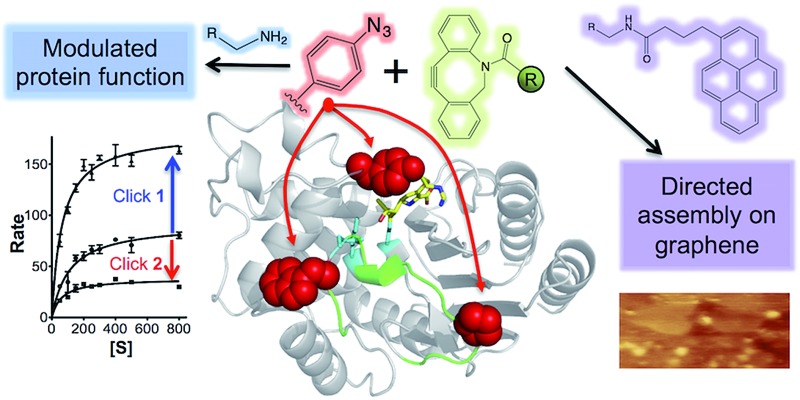
Designed phenyl azide incorporation combined with bioorthogonal Click chemistry to regulate enzyme activity, or promote its stable assembly on graphene.

## Introduction

Post-translational modification (PTM) is central to biology by expanding and modulating the function of a large number of proteins.^1^ Many of these events essentially permanently covalently modify a protein, ranging from attachment of small moieties (*e.g.* methylation,^2^ cofactors^3^) to larger events such as proteolysis and glycosylation. Each of these factors can impact significantly on protein structure and function thus influencing and even enabling inherent protein activity. The presence of sequence and/or structural motifs in combination with of a wide variety of subsidiary machinery, mostly enzymes, is required to achieve exquisite specificity both in terms of the target protein and the spatial position in the modified target. The complexity of these systems can be a significant hindrance with respect to their transfer to new proteins where such PTMs are not inherent. Furthermore, PTM is largely restricted to a select chemical set preexisting in nature. Precise modification with non-natural adducts may be a more appropriate and useful means to expand and modulate protein function,^4,5^ including for use in non-biological contexts.

Covalent modification with non-natural adducts is traditionally achieved using chemistry inherent to the natural amino acid repertoire, mostly amine, carboxyl and thiol groups. The main problem is lack of specificity as such chemistry is normally distributed across the surface of a protein and is ubiquitous in the proteome. A more general and powerful approach is the introduction of new chemically reactive handles not present in the native 20 amino acid set through the use of an expanded genetic code^6^ (see ref. 7–10 for recent reviews). The unique reactivity of a chosen non-natural amino acid (nAA) together with the ability to select both the target protein and the residue within the target means that specificity comparable to natural PTM events can be achieved.^10,11^ The use of highly specialized caged nAAs has been very effective in controlling activity^12–15^ but is restricted to certain protein types and chemistry, and can add significant bulk to the amino acid side chain during the production and folding of the nascent polypeptide and its subsequent folded form.

The incorporation of phenyl azide chemistry into proteins through the use of *p*-azidophenylalanine (azF)^17^ is an attractive alternative. As well as being only one atom bigger than the natural amino acid tyrosine and having been successfully incorporated into a wide variety of proteins,^7–9^ the phenyl azide moiety opens up different routes to non-natural PTM (nnPTM): photochemical transformations and Click chemistry adduct addition.^9^ The use of phenyl azide photochemistry to control protein activity has recently been demonstrated.^18–22^ Azide–alkyne cycloaddition is fast becoming a useful approach for orthogonal biomolecule conjugation but has largely been used in a passive way, for example, to label proteins.^7,23–25^ Given the versatility in terms of the array of adducts available coupled with the inherent bioorthogonality and biocompatibility,^26^ it is surprising Click chemistry has not been used more extensively as a general direct modulator of protein activity. Additionally, useful adducts can be placed at strategic positions to expand and facilitate protein function in a manner akin to co-factors. This includes attachment of entities to facilitate interfacing and assembly with secondary systems or materials.^27–31^ As in many natural biomolecular assemblies, a defined and optimal protein–material interface is critical for maximal communication between the individual elements. Interfacing proteins with carbon sp^2^ materials such as graphene is gaining significant interest as it forms the basis for constructing hybrid bio-transistors in which events at even the single protein molecule level can be used to gate conductance through graphene.^27,32^

**Fig. 1:**
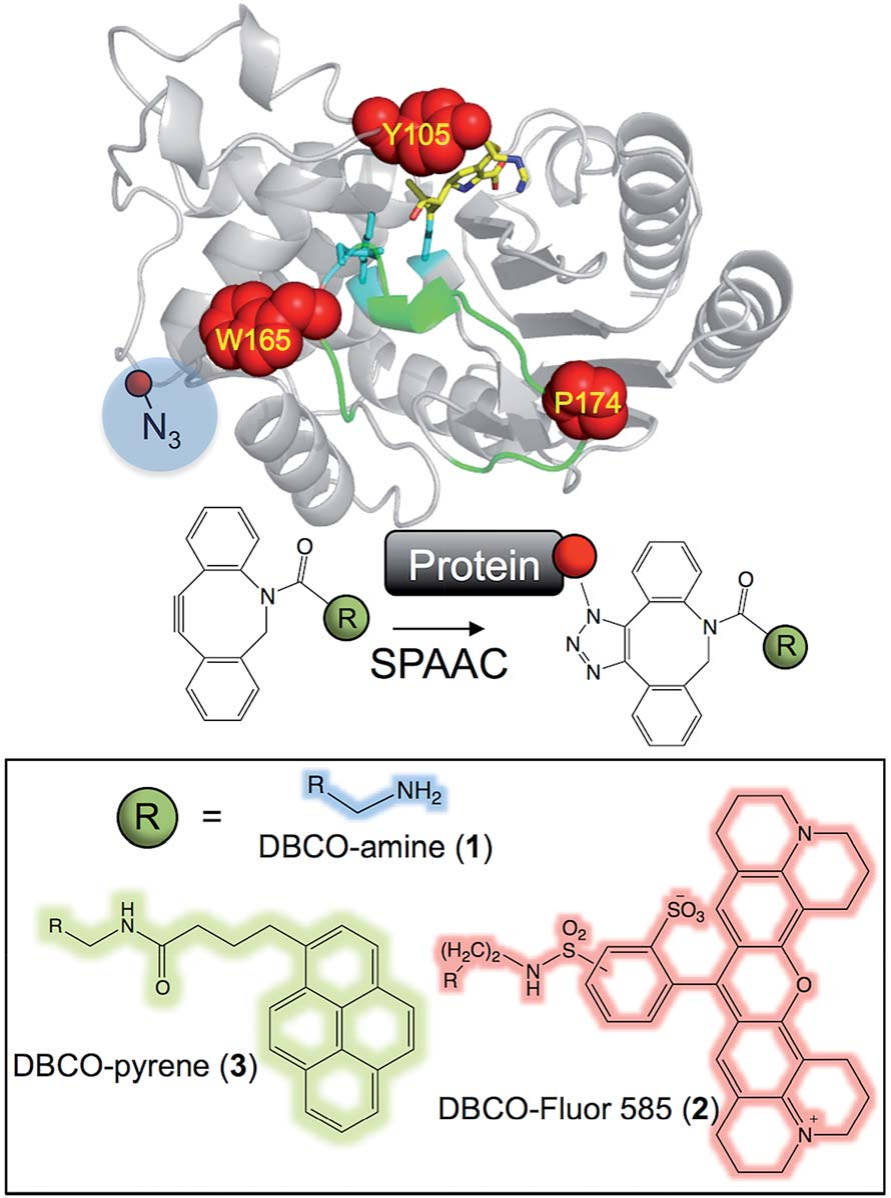
Modification of TEM β-lactamase. The structure of TEM β-lactamase (top left) with the inhibitor imipenem (yellow sticks to emphasise the substrate binding pocket) highlights the residues targeted for replacement with azF (red spheres) together with the key catalytic residues (cyan sticks) and the Ω-loop (green). The protein structures were generated using PyMol.^16^ A representation of the SPAAC reaction using the activated alkyne present in the core DBCO moiety is shown (top right). The R groups attached to the DBCO used in this study are shown (bottom half).

Here, we show that the activity and assembly onto pristine graphene of the antibiotic resistance protein TEM β-lactamase^33,34^ can be directly controlled by Click chemistry. By genetically encoding phenyl azide chemistry at designed positions in TEM and using Cu-free biocompatible strain promoted azide–alkyne cycloaddition (SPAAC; Fig. 1)^35,36^ different adducts can be attached (**1**, **2** and **3**; Fig. 1) that either reduce or restore activity on modification, and define assembly of TEM on graphene.

## Results and discussion

The three main adducts with a core dibenzylcyclooctyne (DBCO) reactive handle (Fig. 1) chosen have very distinct properties: **1** is an amine derivative of DBCO that has hydrogen donor and acceptor groups opening up the potential to form H-bonds between residues not normally close enough to each other in the protein structure; **2** is a large, planar and hydrophobic rhodamine dye (Texas Red) that has proved useful in labelling proteins for fluorescent imaging^23^ but could also act as an effective bulk spatial and steric blocking element if required; **3** is a pyrene derivative that will aid protein interfacing with extended carbon sp^2^ materials (*e.g.* graphene) through π–π stacking.^29,37^ Both **1** and **2** are “off-the-shelf” products while **3** can be generated by a simple succinimidyl ester reaction. Thus, all **3** adducts can easily be accessed by the wider bioscience community without any synthetic chemistry knowledge. The azF dependent production of active enzyme is shown in ESI Fig. 1.†

### Modulating enzyme activity

**Fig. 2:**
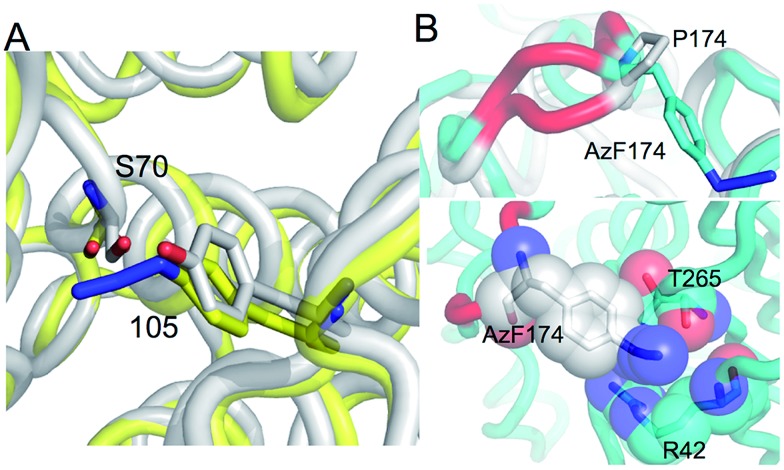
Molecular design models of TEM azF variants. (A) Model of TEM^Y105azF^ (yellow) aligned with wt TEM β-lactamase (grey; PDB ; 1btl), with the active site S70 shown as stick representation. (B) Alignment of the TEM^P174azF^ (cyan) with wt TEM (grey; PDB ; 1btl). The region highlighted in red covers residues 173–175. (C) Interaction of AzF174 with neighbouring residues.

**Fig. 3:**
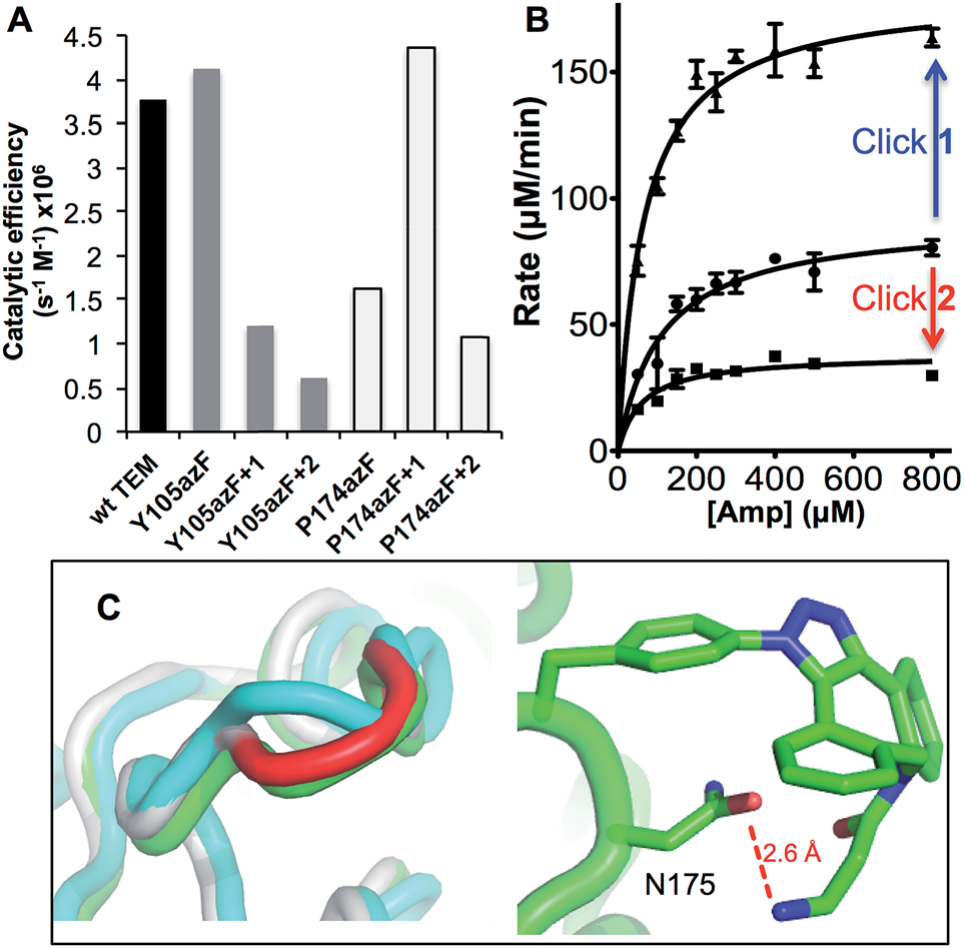
Enzyme kinetics of ampicillin hydrolysis by TEM β-lactamase variants pre- and post-Click modification. (A) Catalytic efficiency of each variant before and after modification with **1** (+1) or **2** (+2) derived from *K*_M_ and *k*_cat_ (ESI Table 1†). (B) The effect of modifying TEM^P174azF^ with **1** (Click 1) or **2** (Click 2) on Michaelis–Menten kinetics. (C) Modelled changes to backbone structure of TEM^P174azF^ (cyan) on modification with **1** (green) compared to wt TEM (grey) (top panel) and the configuration and local interactions (hydrogen bond in red) of **1** (bottom panel).

The *in silico* design process was performed using a combination of ROSETTA^38^ and molecular dynamics^39^ as outlined in the Supporting Methods.† Y105, a partially surface exposed residue (∼110 Å^2^ ≈ 50% relative surface exposure), was chosen as a site for negative modulation through nnPTM due to its location close to the catalytically important SDN loop and its role in forming a partial lid over substrate binding cleft (Fig. 1; ESI Fig. 2†). Y105 is thought to be especially important in defining the size of the substrate binding cleft (therefore substrate specificity)^40^ and local dynamics of residues critical for activity.^41^ *In silico* modelling predicted exchanging the hydroxyl group for an azide should not have a major effect on enzyme structure around the locality of residue 105 (Fig. 2a). In fact ampicillin hydrolysis was slightly enhanced (higher *k*_cat_) for TEM^Y105azF^ but had a slightly lower affinity (higher *K*_M_) making overall catalytic efficiency similar to wt TEM (Fig. 3 and ESI Table 1†). TEM^Y105azF^ was receptive to modification by SPAAC as indicated by the estimated labelling efficiency of ∼80% with **2**.

As predicted, TEM^Y105azF^ was largely inhibited on modification with either **1** or **2** (Fig. 3) despite neither having any significant inherent inhibitory effect on wild-type TEM (ESI Table 1†). Both catalysis and substrate binding were disrupted as evident by the decrease in *k*_cat_ and increase in *K*_M_ (ESI Table 1†) for ampicillin resulting in an overall decrease in catalytic efficiency of ∼70% and 85% when modified with **1** or **2** respectively compared to unmodified protein. This is similar to the most deactivating site-directed mutants of Y105.^40^ Modelling of the covalent complex (*vide infra*) between TEM^Y105azF^ and **1** suggested that modification blocks access at one end of the substrate binding site, with the amine of **1** forming a hydrogen bond with the backbone carbonyl group of G238 (ESI Fig. 3†). The enzyme kinetics suggests that simple steric blocking of substrate binding is unlikely to be the sole mechanism of action as catalysis and substrate binding are equally affected, similar to that observed for classical mixed inhibition models. The relatively small difference in catalytic efficiency of TEM^Y105azF^ modified with either **1** or **2** indicates that the most significant effect is occurring close to the linkage site, as suggested by the covalent complex model (ESI Fig. 3†). However, the bulkier group of **2** is exerting a slightly greater effect with regards to *k*_cat_ rather than *K*_M_ (Fig. 3 and ESI Table 1†) suggesting that long range interactions made by the rhodamine dye moiety may be forcing the structure around residue 105 to adapt less catalytically proficient form. Currently, the separation of modified from unmodified protein has proved unsuccessful, so the observed activity may be contributed by unmodified protein only (estimated to be ∼20%; *vide supra*). Therefore, the inhibition levels observed here represent the lowest currently achievable and we cannot rule out modified species are essentially inactive.

P174 is another largely surface exposed residue (∼80 Å^2^ ≈ 60% relative surface accessibility) that is relatively distant from the active site (Fig. 1). P174 however contributes towards one of the turns that comprise the Ω loop (Fig. 1 and ESI Fig. 4†), a region critical for substrate binding, substrate specificity and catalysis.^42,43^ It was hypothesized in the design process that mutating P174 to a non-cyclic amino acid would result in local conformational changes around the turn region. Molecular modelling supported this hypothesis with the structure of the turn slightly shifted compared to wt TEM (Fig. 2b and ESI Fig. 5†). The side chain of azF174 lies approximately perpendicular to the native proline, with the azido moiety fitting into a shallow pocket and making a local polar interaction network with R42 and T265 (Fig. 2c). These new interactions may act as the driver of local conformation changes associated with the P174azF mutation, which in turn alters the local bonding network that subtly shifts the conformation of both the active site and substrate binding residues, including S70 (ESI Fig. 5†). Based on the TEM^P174azF^ model it was further postulated that on modification with **1**, the formation of the triazole link would break the interaction network with R42 and T265 so reconstituting the original loop structure and activating the protein. The amine group also has the potential to form new long range hydrogen bonds in spatially local regions (Fig. 3 and ESI Fig. 4†).

As suggested by the model, replacement of P174 with azF results in a significant change in activity. The overall catalytic efficiency of TEM^P174azF^ was 60% lower compared to wt TEM (Fig. 3). The major effect was on *k*_cat_ which was 3 fold lower compared to wt TEM (ESI Table 1†), supporting the evidence from the model that the likely effect of azF incorporation at residue 174 is to disrupt interactions of residues associated with catalysis. This was offset by a slightly increased ampicillin affinity.

TEM^P174azF^ was accessible to Click modification, but efficiency was lower (estimated ∼33% using the absorbance properties of **2**) compared to TEM^Y105azF^. It is unknown how local protein microenvironment dictates SPAAC efficiency but dynamics, relative exposure to aqueous solvent and the character of shallow “pockets” have been suggested as possible determinants.^23^ The molecular model suggests that interaction of the azide moiety with other residues, which may play a role through, for example, altering the relative populations of resonance structures sampled by the azide moiety (R–N=N^+^ =N^–^/R–N^–^–N^+^ ≡N), and/or its accessibility and available orientations to the incoming DBCO. The effect of the SPAAC nnPTM on the ability of TEM^P174azF^ to hydrolyse ampicillin varied depending on the DBCO adduct. Modification with **2** exerted a similar but somewhat smaller inhibitory effect (∼40% drop) as observed for TEM^T105azF^ (Fig. 3 and ESI Table 1†). Modification with **1** resulted in a significant increase in overall activity restoring apparent catalytic efficiency essentially to wild-type levels (Fig. 3). This should be considered as a lower estimate of activation as ∼70% of the protein may be unmodified (*vide supra*). With both adducts, the most significant contribution was the change in *k*_cat_ of TEM^P174azF^ compared to unmodified protein (ESI Table 1†); modification with **1** increased *k*_cat_ by 210% while addition of **2** reduced *k*_cat_ by almost half.

To understand how **1** exerts its beneficial effect on TEM^P174azF^, the nnPTM product was modelled. Using the TEM^P174azF^ model as a starting point, the triazole linkage between azF and DBCO of **1** was generated *in silico* by parameterizing the azF–DBCO complex to calculate optimized geometries and the electrostatic potentials. The model was subjected to molecular dynamics for a total of 5 ns. The model of nnPTM product, termed TEM^P174azF^+**1** suggested that the general structure was closer to wt TEM than the original TEM^P174azF^ model (Fig. 3c and ESI Fig. 5†). The Ω-loop structure around residue 174 and the local bonding networks for TEM^P174azF^+**1** were largely comparable with wt TEM. The orientation of residue 174 differs significantly to accommodate **1** (ESI Fig. 5†). The amine group of **1** bends back and appears to come within hydrogen bonding distance (∼2.6 Å) of the side-chain carbonyl group of the adjacent residue N175 (Fig. 3c). The loss of the azide group on formation of the trizole removes the interactions with R43 and T238. However, the reason for **1** acting as an activator and **2** as an inhibitor may be down to the reduced bulk and ability of **1** to form local H-bonds.

### Protein–graphene interfacing

In some instances, it would be attractive to select a residue intimately associated with active site regions so that secondary non-related events can be coupled to activity without significant effect on function. Linking proteins to carbon sp^2^ materials such as graphene is especially attractive; local charge and electrostatic changes in the protein can be used to “gate” the electronic properties of the sp^2^ material^27,44^ so generating sensing systems with ultimate single molecule resolution. The pyrene moiety of **3** (Fig. 1) is especially attractive as an interfacing agent as it allows defined coupling of a protein to the sp^2^ material through π stacking.^37^ This has been demonstrated previously using optimally placed cysteine residues as the reactive handle for attachment to pyrene-coated single walled carbon nanotubes.^27^ However, this required removal by mutagenesis of native cysteine residues. Here, we demonstrate that defined enzyme interfacing on a clean graphene face is feasible though the use of designed azF placement.

**Fig. 4:**
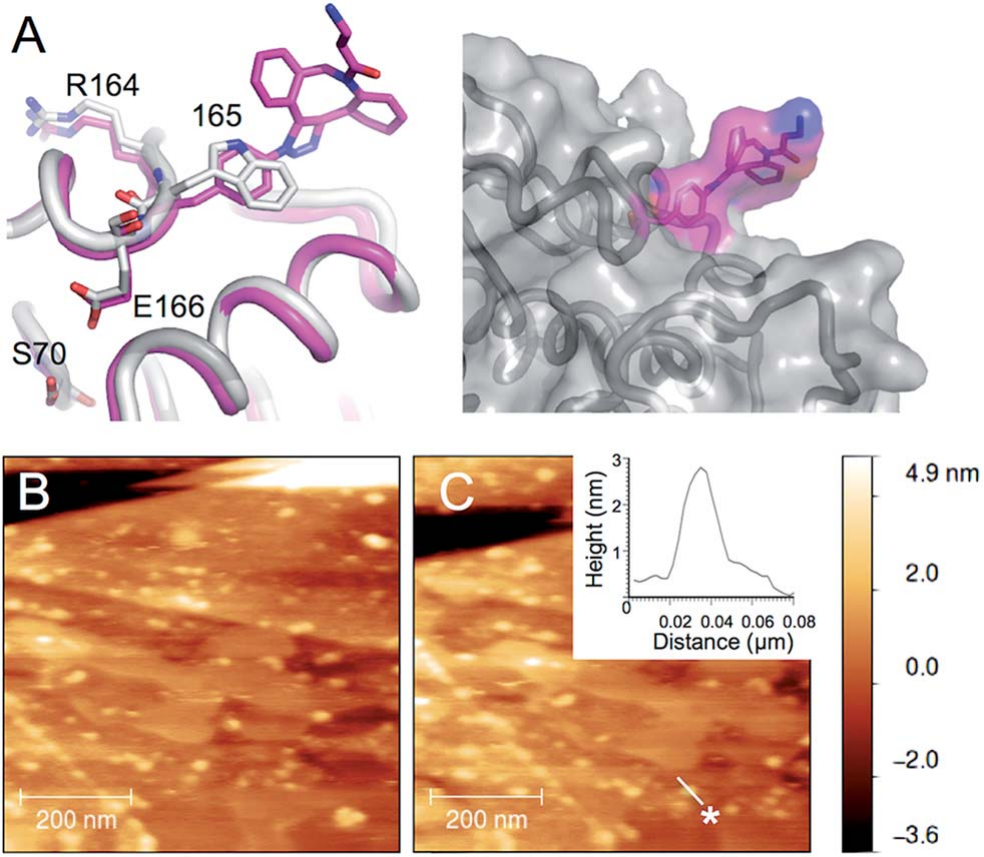
AFM imaging of pyrene-modified TEM^W165azF^ on graphene. (A) Model of TEM^W165azF^ (magenta) modified with **1** aligned with the crystal structure of wt TEM (grey) with the residues either side of residue 165 and the catalytic S70 highlighted. The surface representation of TEM^W165azF^ modified with **1** (left panel) illustrates that the Click adduct points out and away from the protein. Repeated AFM imaging of the surface after the (B) sixth and (C) tenth scans shows that the proteins bind stably to the surface. The height analysis of a single TEM^W165azF^-**3** molecule, which is selected in C (highlighted as *) is shown as an inlay to C. ESI Movie 1† provides a full trace for the imaged area above over 10 scans.

W165 is another partially surface exposed residue (∼100 Å^2^ ≈ 50% relative surface accessibility) that fulfils the required criteria (Fig. 1). It resides in the Ω-loop and splits two functionally important residues; E166 is a conserved catalytic residue involved in proton shuttling^34^ and R164 forms buried ionic interactions critical for stabilizing the Ω loop and thus the positioning of E166.^45^ The approach used to model TEM^P174azF^ modified with **1** was applied to the W165azF mutation to ascertain the potential position of the adduct group and its effect on structure (Fig. 4a). Modelling suggested that the overall structure of the protein would be largely unperturbed and the adduct would point outwards into the solvent. Mutating W165 to azF did slightly reduce overall catalytic efficiency towards ampicillin (1.5 fold) but crucially, modification with **3** was feasible and had little effect on overall activity (ESI Fig. 6†).

By coupling **3** to TEM^W165azF^ prior to surface assembly we can directly interface the protein with graphene in a defined manner without surface coating with pyrene, as has been previously used.^27,46^ Intermittent-contact mode AFM (tapping mode) imaging indicated that TEM^W165azF^ modified with pyrene binds stably to graphene surfaces (Fig. 4b and c; ESI Fig. 7 & Movie 1†). The protein molecules are not significantly disturbed by multiple scans. The average apparent heights were 3 nm, which is close to the predicted height of the protein bound in the designed orientation (∼3 nm; ESI Fig. 7†). The average apparent lateral dimension was larger than predicted (10 nm *versus* 5 nm), as a result of tip convolution effects. In comparison, wt TEM did not stably bind to the surface, with tip contamination commonly observed after multiple scans (ESI Fig. 8†).

## Conclusions

SPAAC is a powerful approach for precise and designed protein PTM that supplement protein activity using a non-intrusive, non-caged reaction handle. We have demonstrated here that SPAAC can be used beyond simple passive labelling of proteins and can actively modulate activity through different mechanisms without the need to synthesize specialized molecules. It also facilitates the interfacing of proteins in a designed and defined manner with useful active materials such as graphene. This negates the need to remove inherent chemistry through mutagenesis allowing the focus to be on placing the reactive handle for optimal coupling between the protein and material. *In silico* modelling with both nAA and adducts can greatly aid the design process by which useful variants are generated and their affects rationalised, which will in turn lead to more accurate designs.

## Experimental

### *In silico* protein modelling

A detailed description of the *in silico* modelling of TEM containing the azF mutation and modification with the DBCO-amine adduct (**1**) are provided in the ESI.† Briefly, geometry optimised structure files, force field parameters for azF and azF–DBCO amine adduct complexes were generated. The published crystal structure of TEM β-lactamase to 1.8 Å resolution (PDB code ; 1BTL^45^) was used as a starting point for Monte Carlo simulations within the ROSETTA software package.^38^ The lowest energy model was used as the starting point for molecular dynamics using GROMACS.^39^ The modelling was run on the Raven cluster as part of the Advanced Research Computing @ Cardiff facility.

### Enzyme activity assays

The wt and mutant TEM variants were generated as outlined in the ESI.† The proteins were expressed in *E. coli* using a bespoke plasmid based on pBAD called pBADKAN, and purified as described in the ESI.† The TEM-dependent kinetics of ampicillin hydrolysis (*ε*_235_ = 1500 M^–1^ cm^–1^) were determined spectrophotometrically using 1 cm path length QS quartz cuvette (Hellma). Hydrolysis assays were carried out in a 1 mL reaction volume. Purified enzyme was diluted to a final concentration of 250 ng μL^–1^ in 50 mM sodium phosphate buffer, pH 8 at room temperature. Reactions were started by addition of ampicillin and hydrolysis was measured by the decrease in absorbance at 235 nm. Ampicillin concentrations ranged from 50 μM to 800 μM. Kinetic parameters were calculated using initial rate of hydrolysis at each substrate concentration and then fitting to the Michaelis–Menten equation using GraphPad Prism. TEM β-lactamase activity using the colorimetric substrate nitrocefin was performed essentially as described previously.^47,48^

### Click modification

SPAAC reactions were performed on pure protein using a five-fold molar excess of dibenzylcyclooctyne (DBCO) reagent to protein. DBCO-amine (**1**) and DBCO–Fluor 585 (**2**) were obtained from Click chemistry tools, while DBCO–pyrene was synthesized from DBCO-amine and 1-pyrenebutanoic acid, succinimidyl ester *via* nucleophilic substitution. Reactions were left overnight at room temperature in PBS. SPAAC reactions with **2** were analysed by SDS-PAGE and subsequent imaging of the fluorescent dye on a transilluminator. Labelling efficiencies were calculated as described in the ESI.†

### Protein deposition on graphene and AFM imaging

Protein samples were deposited onto graphene on a copper foil substrate. Monolayer graphene was deposited on the copper substrate by chemical vapor deposition (CVD) using methane as carbon source. A detailed description of the growth procedure is provided in the ESI.† The foil pieces (∼4 mm^2^) were immersed in PBS containing 1 nM protein and incubated at room temperature for 10 min. Following incubation, the foil pieces were rinsed with high purity deionised water and dried with N_2_ gas. AFM imaging was performed in using a Veeco Nanoscope IIa (Bruker) in tapping mode. The same area was scanned up to 10 times with a total of 6 different regions imaged to check the stability of the protein molecules on the surface of graphene. A video of the surface scans is provided as ESI.† Although the images show some drift, it is clear that the proteins are stably bound to the surface.
