# Correction: Functional modulation and directed assembly of an enzyme through designed non-natural post-translation modification

**DOI:** 10.1039/c6sc90025a

**Published:** 2016-04-21

**Authors:** Andrew M. Hartley, Athraa J. Zaki, Adam R. McGarrity, Cecile Robert-Ansart, Andriy V. Moskalenko, Gareth F. Jones, Monica F. Craciun, Saverio Russo, Martin Elliott, J. Emyr Macdonald, D. Dafydd Jones

**Affiliations:** a School of Biosciences , Cardiff University , Cardiff , UK . Email: jonesdd@cardiff.ac.uk; b School of Physics and Astronomy , Cardiff University , Cardiff , Wales , UK; c Centre for Graphene Science , University of Exeter , Exeter , Devon , UK

## Abstract

Correction for ‘Functional modulation and directed assembly of an enzyme through designed non-natural post-translation modification’ by Andrew M. Hartley *et al.*, *Chem. Sci.*, 2015, **6**, 3712–3717.



## 

In the original manuscript, the scan numbers for parts B and C of Fig. 4 were given incorrectly in the figure caption. The corrected description of parts B and C should read: “Repeated AFM imaging of the surface after the (B) 11th and (C) 16th scans shows that the proteins bind stably to the surface”.

The Royal Society of Chemistry apologises for these errors and any consequent inconvenience to authors and readers.

