# Supplementary material for: Functional modulation and directed assembly of an enzyme through designed non-natural post-translation modification
Source: Chem Sci. 2015 Mar 31;6(7):3712–7. doi: 10.1039/c4sc03900a (PMC5496188; doi:10.1039/c4sc03900a)
Supplement: Supplementary file 1 [file SC-006-C4SC03900A-s001.pdf]

Electronic Supplementary Material (ESI) for Chemical Science.
This journal is © The Royal Society of Chemistry 2016

Functional modulation and directed assembly of an enzyme through designed non-natural post-translation modification.

Andrew M Hartley, Athraa J Zaki, Adam R McGarrity, Cecile Robert-Ansart, Andriy V Moskalenko, Gareth F Jones, Monica F Craciun, Saverio Russo, Martin Elliott, J Emyr Macdonald & D. Dafydd Jones.

08 March 2016

Note added after first publication: This Supplementary Information file replaces that originally published on 05 April 2015. In the original file, parts (a) and (b) of Supporting Figure 8 were reversed.

Supporting Information

Supporting Methods.

Mutant generation and protein production. The original pBAD plasmid (Life Technologies) was altered so as to replace the normal ampicillin *bla* selection marker gene with kan^R kanamycin resistance. With the pBAD vector as template, primers annealed to either side of the Amp^R gene and PCR using the Phusion system (NE Biolabs) was used to amplify the basic backbone of the vector minus *bla*. Digestion of the PCR product with NotI and SalI generated complimentary sticky ends for Kan^R insertion, generating the plasmid pBADKAN. The *bla* gene encoding TEM β-lactamase was amplified from pUC18 and cloned between the NcoI and KpnI sites and under the control of the P_{BAD} promoter generating a new plasmid termed pBLAD (pBADKAN plus *bla* gene). The TAG mutations were introduced at specific positions in the *bla* gene by site-directed mutagenesis using the Phusion DNA polymerase system (NE Biolabs).

E. coli Top10 cells transformed with the two required plasmids (pBLAD and pDULE containing the tRNA and the amino-acyl cyanophenylalanine tRNA-synthetase [1, 2]) were grown in a 5 mL LB starter culture overnight. The starter culture was diluted 1/200 and used to inoculate 50 mL autoinduction media [3, 4] supplemented with 10 μg/ml kanamycin and 10 μg/ml tetracycline. For TEM variants containing a TAG mutation, 1 mM *p*-azido-L-phenylalanine was added to the culture before

incubation. Cultures were incubated at 37°C in a shaking incubator (200 rpm) for 24 hours. Cultures were grown in the dark to prevent photolysis of the phenyl azide group. Protein was purified from lysed cells by nickel affinity chromatography using a HisTrap column (GE Healthcare) connected to an AKTApurifier 10 (GE Healthcare). Cell lysates were spun at 17000 x *g* and the soluble fraction was loaded onto the column equilibrated with PBS (100 mM sodium phosphate, 300 mM NaCl, pH 8). An imidazole (Fisher) gradient from 0 mM to 250 mM (in PBS) was then applied to the column over 20 column volumes to elute protein. Eluted protein was measured for absorbance at 280 nm and presence of protein was confirmed using SDS-PAGE and nitrocefin activity assays. For small-scale protein production, His SpinTrap columns (GE Healthcare) were used to purify protein after cell lysis. Proteins were finally dialysed into PBS to remove imidazole.

Modification efficiency was determined using the molar absorbance coefficients of the protein (24000-29500 M^{-1} cm^{-1}at 280 nm) and **2 (**DBCO-585 dye [97000 M^{-1} cm^{-1} at 595 nm]). Protein (both modified and unmodified) was separated from free **2** using centrifugal filters with a molecular weight cutoff of 10000 Da. The theoretical molar absorbance coefficient of TEM β-lactamase was calculated using ProtParam (http://web.expasy.org/protparam/) with azF considered as a tyrosine. These values were used to determine the concentration of total protein and modified protein in the sample by comparing the absorbance ratio at 280 nm [protein] and 595 nm [adduct] to determine the final modification efficiency.

Graphene fabrication. Monolayer graphene films were grown on 25μm thick Cu foils via chemical vapour deposition (CVD) in a Moorfield nanoCVD-8G cold-wall furnace. Cu foils were first annealed in a hydrogen atmosphere at 1035°C. Growth of graphene on the foil surface was then initiated in the presence of CH_4 and H_2 at 1000°C with pressure increasing from zero to 0.7 Torr. Once the growth phase was completed, the furnace chamber was flushed with Ar gas and cooled to room temperature.

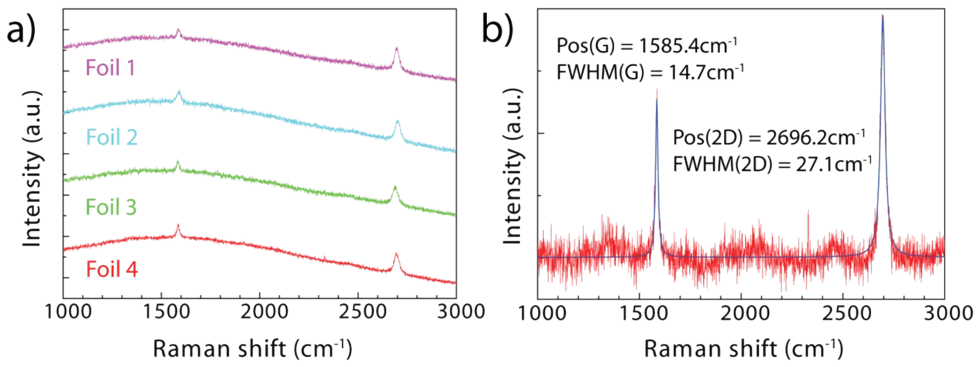

Raman spectra of monolayer CVD graphene on Cu foils. a) Raw data acquired at a random location of four separate foils. Intensities of foil spectra have been offset to allow easy comparison. b) Foil 4 after baseline subtraction with a single lorentzian fit on both the G and 2D peaks.

Evidence for the presence of monolayer graphene was provided using Raman spectroscopy measurements (see Figure above). Raman spectra were collected in air at room temperature using a Renishaw spectrometer with a λ=532nm excitation laser focused to a spot size of 1.5μm diameter by a 100x objective. The incident beam power was kept at 3.4 mW to avoid thermal damage to the graphene films. The figure above (panel a) shows Raman spectra obtained at a random location on 4 different Cu foils after CVD growth of graphene. The G and 2D peaks, signatures of sp^2 hybridized carbon [1], are clearly present in all cases. In order to analyse peak locations and film quality, the baseline of raw data was corrected, leaving just the sp^2 carbon peaks. The figure above (panel b) shows a typical Raman spectrum after baseline subtraction using a polynomial fit. The G and 2D peaks are each fitted with a single Lorentzian where the relative peak heights are given by an I(2D)/I(G) ratio of 1.54 and a full width half maximum of 27.1 cm^{-1} for the 2D peak. The combination of these characteristics implies that there is no interaction between AB-stacked carbon

atoms present and the film is therefore monolayer [2]. A D peak, resonance of breathing modes in sp^2 rings adjacent to defects, around 1350 cm^{-1} was not resolvable in any of the foil spectra. This suggested the graphene films possessed long-range order with relatively few defects or absent atoms in the lattice.

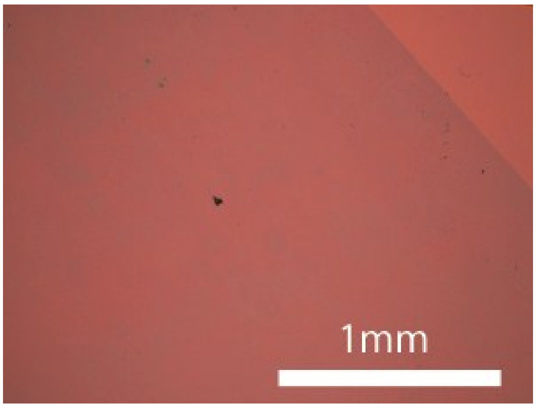

Optical microscope image of monolayer CVD graphene transferred onto an SiO2/Si substrate using ammonium persulfate etchant and PMMA-assisted transfer.

To test film continuity, a CVD-grown graphene film was transferred onto an SiO_2/Si substrate using standard PMMA-assisted transfer techniques and examined under an optical microscope, as shown above. Macroscopic film continuity was demonstrated by the absence of scrolled clusters, a typical result of transferring of discontinuous films [3], and the absence of cracks over several square millimetres. Contaminants on the graphene film were residual traces of PMMA and etchant compounds.

***In silico* modeling.**

The *in silico* protein design and modelling was broken down into two main steps: initial design using ROSETTA [5] followed by molecular dynamics. Structure files for azF and azF-DBCO adduct complexes were made using Avogadro [6]. Force field parameters were derived based on the electrostatic model approach, which uses electrostatic potential (ESP) to describe the electronic structure of nAAs and complexes [7]. Geometry optimization and ESP calculations were performed using GAMESS-US [8] at the HF/6-31G* level in order to be consistent with the AMBER99sb force field [9]. The published crystal structure for TEM β-lactamase (PDB code: 1BTL) was used as the starting point for Monte Carlo simulations within the ROSETTA software package [5]. The scoring function was altered according to the

method described previously [10] to include parameters for azF. A representative output from ROSETTA is shown below for $TEM^{Y105azF}$ alone (panel A) and modified with **1** (Panel B). The lowest energy model from 200 Monte Carlo relaxation cycles was used as the starting point for molecular dynamics (MD) simulations.

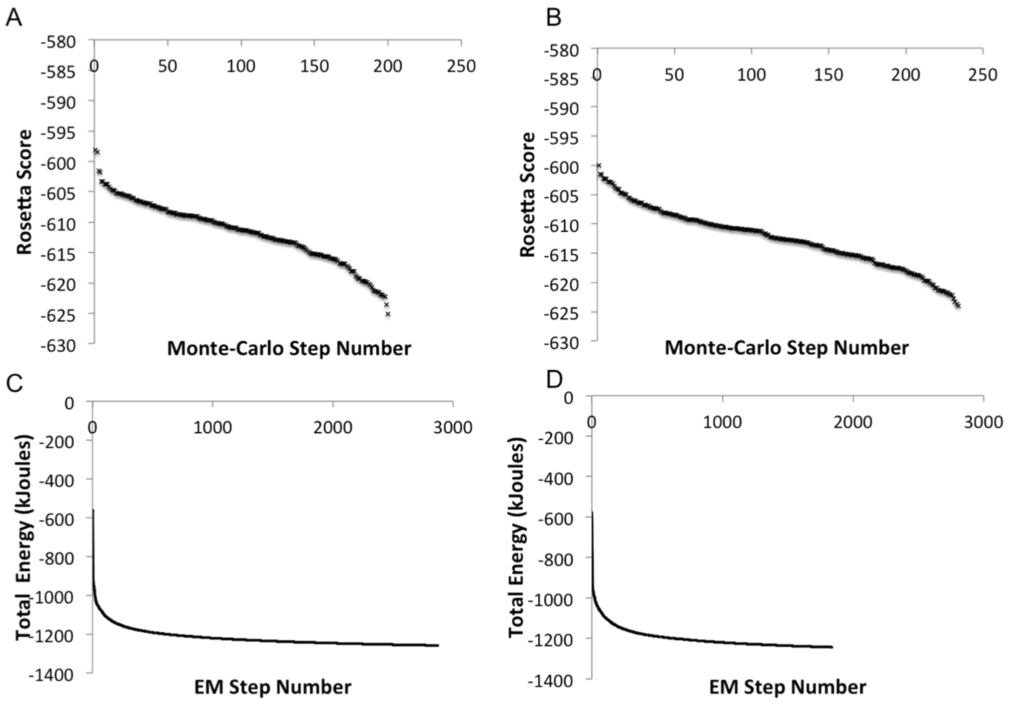

*Overall energy score from Rosetta after Monte-Carlo Relaxation for (A) TEM^{AzF105} after 200 iteration and (B) $TEM\text{-}1^{AzF105}$+**1** after 250 iterations. Total energy profile of TEM in water simulations over energy minimization for (C) $TEM\text{-}1^{AzF105}$ and (D) $TEM\text{-}1^{AzF105}$+**1***

The MD simulations were carried out using the AMBER99sb force field, modified with azF and complex parameters, within the GROMACS software package [11]. The starting structure was placed within a triclinic box with dimensions of 6.4x6.1x7.8 nm. This was populated using the SPC water model to solvate the system to a total number of 8943 solvent molecules. The system was first energy minimised by performing 500 steps of steepest descent method followed by 500 steps of Conjugant Gradient method. The lowest energy state of the system was used as the starting conformation for the molecular dynamics simulation. The simulations were conducted at a constant temperature and pressure of 300 K and 1 atm (NPT). A cutoff of 8 Å was chosen for non-bonded interactions and long range electrostatic interactions were

characterised using the Particle Mesh Ewald (PME) [12]. The simulations were carried out for a total of 5 ns with a time step of 2 fs and the trajectories were analysed using various GROMACS components and visualised using VMD [13] (www.ks.uiuc.edu/Research/vmd/). A representative output from MD is shown above for TEM^{Y105azF} alone (panel C) and modified with **1** (Panel D). For simulations including azF-adduct complexes, the structures were altered using Avogadro to include the complete complex rather than the unmodified nAA prior to energy minimisation then simulations were carried out using the conditions as described above. After 1-1.5 ns, the trajectories reach a plateau suggesting that common structure had been reached, exemplified by TEM^{Y105azF} shown below.

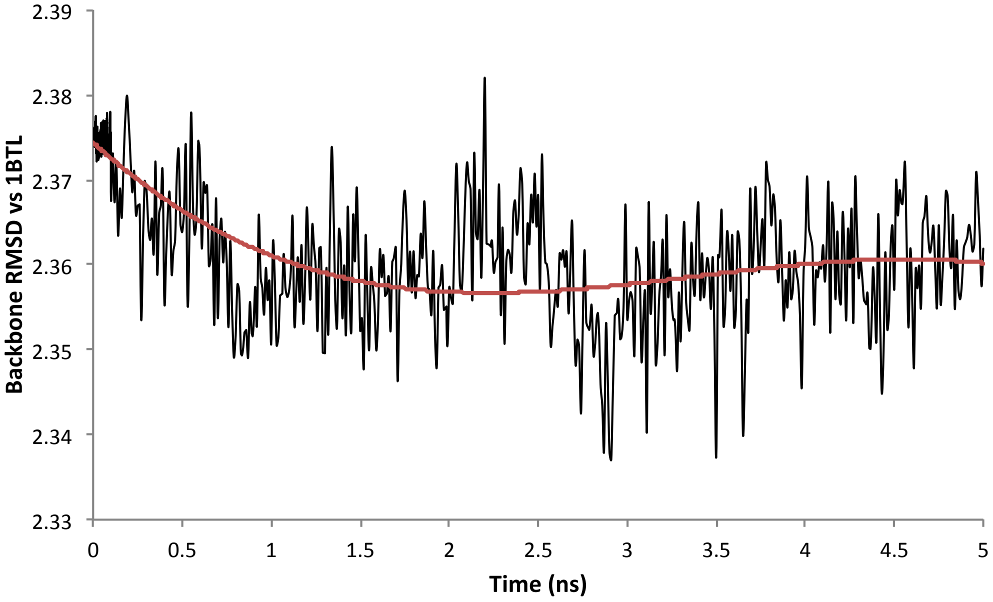

RMSD vs 1BTL crystal structure for the model of TEM-1^{AzF105} over the course of a 5ns simulation. Black line shows RMSD calculated at each time point and Red line shows the running average. This shows that the system stabilizes at 1ns.

Supporting Table 1. Enzyme kinetics of ampicillin hydrolysis by TEM β-lactamase azF variants.

	**wt** TEM			TEM **Y105AzF**			TEM **P174azF**		
	**unmodified**	**Click 1**[a]	**Click 2**[b]	**unmodified**	Click 1[a]	Click 2[b]	**unmodified**	**Click 1**[a]	**Click 2**[b]
k_{cat} (s^{-1})	550.1 ± 20.5	629.7 ± 12.23	481.1 ± 16.96	753.4 ± 16.63	399.04 ± 31.12	194.4 ± 10.58	170.79 ± 13.07	360.31 ± 12.33	74.34 ± 4.17
K_M (μM)	145.9 ± 14.6	128.6 ± 6.51	121.8 ± 12.25	182.7 ± 10.3	332.0 ± 51.16	317.3 ± 34.82	105.5 ± 28.52	82.66 ± 11.38	69.30 ± 17.02
k_{cat}/K_M ($s^{-1}μM^{-1}$)	3.77	4.9	3.95	4.12	1.2	0.61	1.62	4.36	1.07

Click modification with [a] DBCO-amine or [b] DBCO-Fluor 585 as outlined in Figure 1 in the main text.

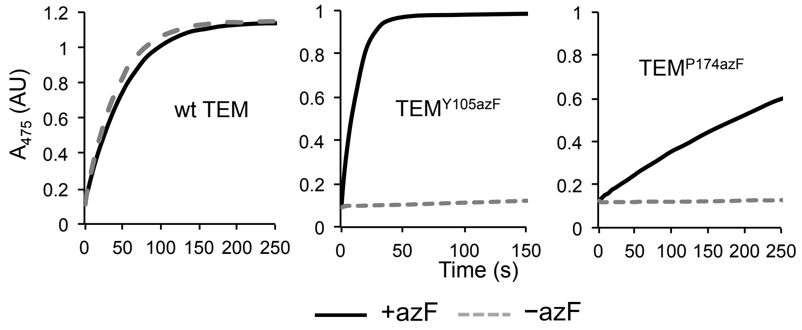

Supporting Figure 1. The azF-dependent production of active wt and variant TEM β-lactamase by *E. coli* as annotated in the figures. β-lactamase Activity of cell lysates in the presence and absence of azF in the cell media. AzF incorporation is determined through amber (TAG) stop codon reprogramming as codons equivalent to residues 105 or 174. In the absence of azF, TAG reverts to a stop codon so truncating the protein at that residue position.

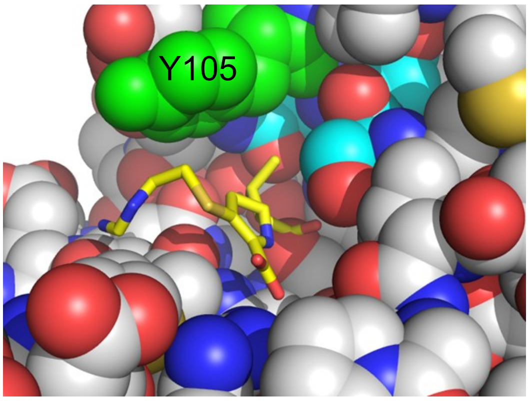

Supporting Figure 2. Position of Y105 in the structure of TEM β-lactamase. The protein structure is shown as spheres, with Y105 coloured green and the SDN loop carbons coloured cyan. The inhibitor IM2 (Imipenem) is shown as yellow sticks. The PDB 1BT5 was used to generate the image using PyMol [14].

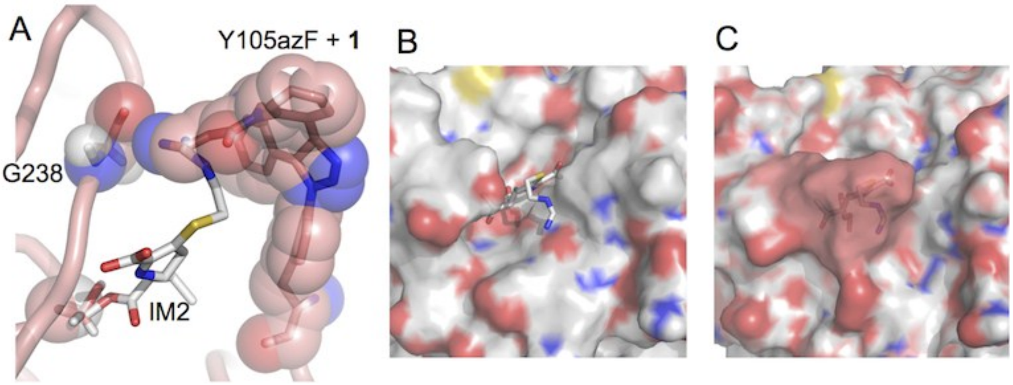

Supporting Figure 3. *In silico* modelling of TEM^{Y105azF} with the DBCO amine (**1**) adduct. (A) Proposed interactions of DBCO-amine with G238 and an active site inhibitor, imipenem (IM2) as observed in the crystal structure 1BT5 [15]. IM2 and **1** will clash sterically due to overlap of the their positions in the active site of TEM. Surface view of (B) TEM-IM2 crystal structure [15] and (C) TEM^{Y105azF}+**1** model. The DBCO-amine moiety is coloured pink in C. Images where generated using PyMol .[14]

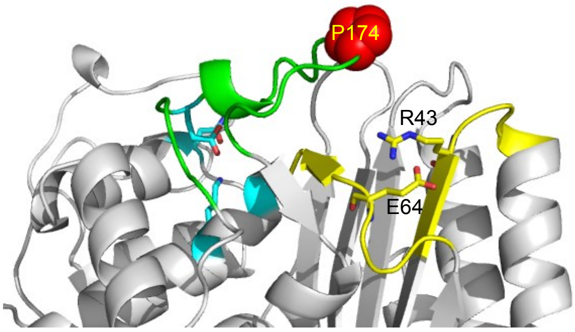

Supporting Figure 4. Position of P174 in the structure of TEM β-lactamase. The protein structure is shown as cartoon, with P174 coloured red, the carbon atoms of key catalytic residues coloured cyan and the Ω loop coloured green. Residues and regions suggested to interact with azF and the nnPTM with **1** are coloured yellow. The PDB 1BT5 was used to generate the image using PyMol [14].

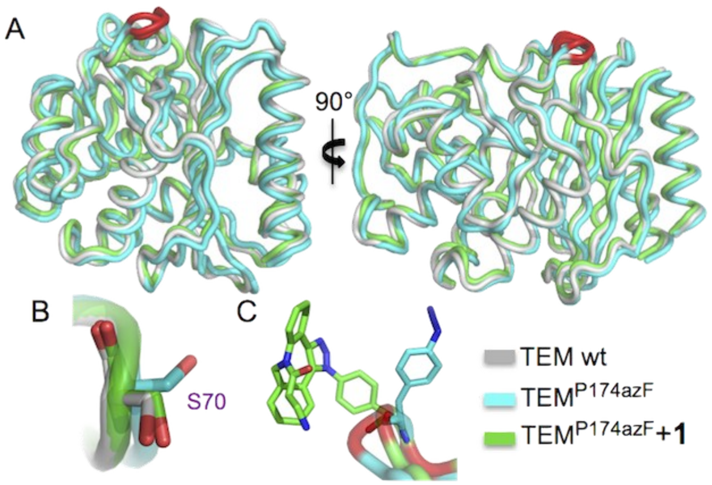

Supporting Figure 5. Comparison crystal structure of TEM β-lactamase [16] with the models of TEM^{P174azF} alone and with **1** attached. Each structure is coloured as indicated in the figure. The region surrounding residue 174 is coloured red. The structural alignment shows: (A) the whole structures; (B) the configuration of the active site serine (S70); (C) the residue 174 region. Images were generated using PyMol .[14].

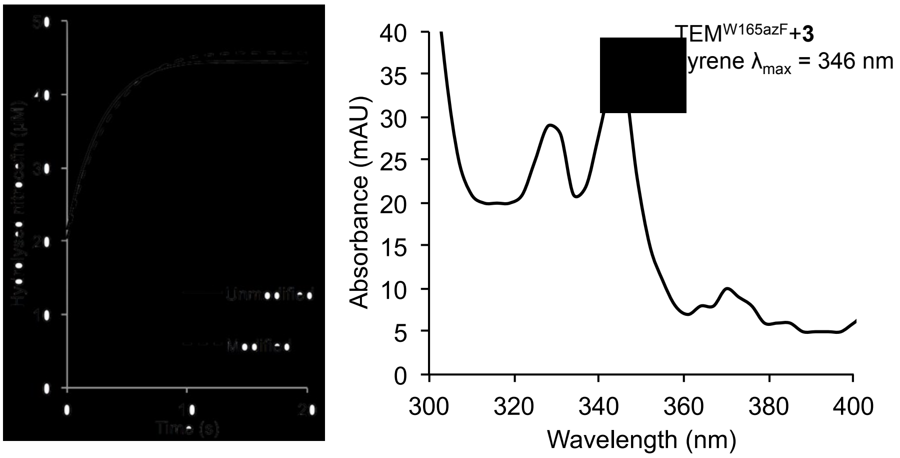

Supporting Figure 6. SPAAC modification of TEM^{W165azF} with **3**. (A) The effect of SPAAC on the activity as determined using the colourimetric substrate nitrocefin used essentially as described previously [17]. Absorbance spectrum of TEM^{W165azF} modified with **3**.

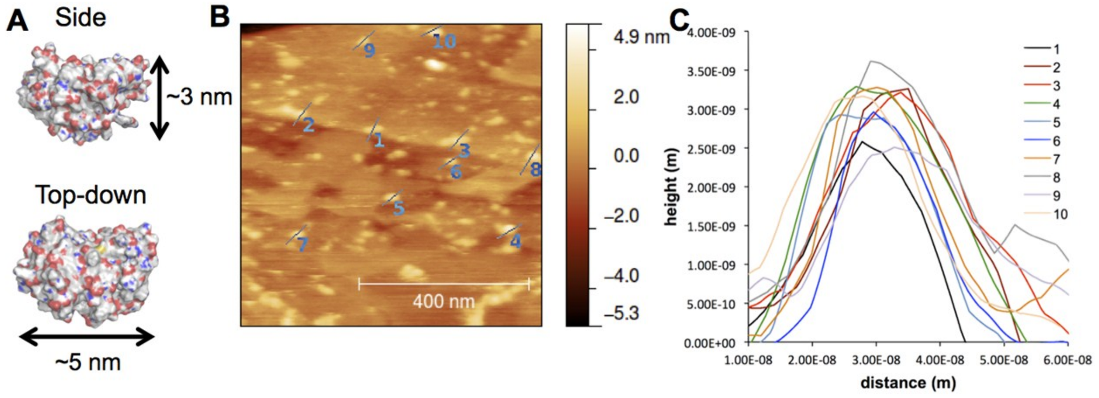

Supporting Figure 7. AFM imaging of pyrene-modified $TEM^{W165azF}$ on graphene. (A) Surface representation of $TEM^{W165azF}$. The idealised height dimensions (side view) and shape (top down) are shown. (B) AFM imaging of graphene surface and (C) the height analysis of a 10 selected areas as indicated in the figure. The areas selected for height measurement analysis were those that when analysed by eye had an approximate shape of the $TEM^{W165azF}$-3 molecule. Supporting Movie 1 provides a full trace for the imaged area above over 10 scans.

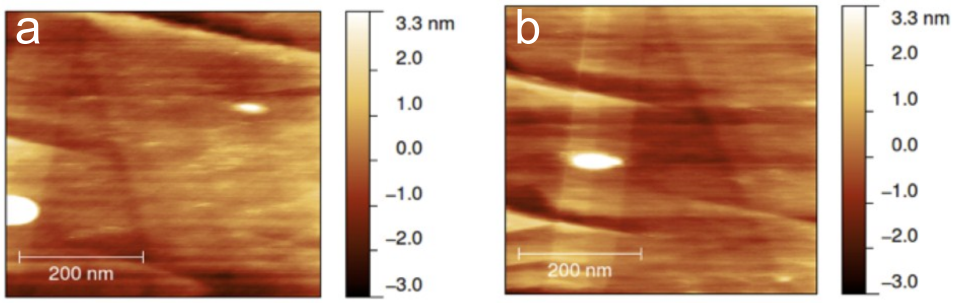

Supporting Figure 8. AFM image of wild-type TEM β-lactamase on graphene. Graphene surfaces were incubated in 1 nM protein solution for 10 minutes. Imaging of the same surface after the (a) first and (b) tenth scan. The protein images are initially poorly defined (presumably due to weak surface binding) become less defined as the proteins are distributed across the graphene surface by the AFM scanning. Imaging clarity is also comprised after multiple scans due to tip contamination.

Supporting References

1. S. J. Miyake-Stoner, C. A. Refakis, J. T. Hammill, H. Lusic, J. L. Hazen, A. Deiters and R. A. Mehl, *Biochemistry*, 2010, 49, 1667-1677.
2. S. C. Reddington, E. M. Tippmann and D. D. Jones, *Chemical communications*, 2012, 48, 8419-8421.

3. F. W. Studier, *Protein Expr Purif*, 2005, 41, 207-234.
4. S. C. Reddington, P. J. Rizkallah, P. D. Watson, R. Pearson, E. M. Tippmann and D. D. Jones, *Angewandte Chemie*, 2013, 52, 5974-5977.
5. K. T. Simons, R. Bonneau, I. Ruczinski and D. Baker, *Proteins*, 1999, Suppl 3, 171-176.
6. M. D. Hanwell, D. E. Curtis, D. C. Lonie, T. Vandermeersch, E. Zurek and G. R. Hutchison, *Journal of cheminformatics*, 2012, 4, 17.
7. C. I. Bayly, P. Cieplak, W. Cornell and P. A. Kollman, *The Journal of Physical Chemistry*, 1993, 97, 10269-10280.
8. M. W. Schmidt, K. K. Baldridge, J. A. Boatz, S. T. Elbert, M. S. Gordon, J. H. Jensen, S. Koseki, N. Matsunaga, K. A. Nguyen, S. Su, T. L. Windus, M. Dupuis and J. A. Montgomery, *Journal of Computational Chemistry*, 1993, 14, 1347-1363.
9. V. Hornak, R. Abel, A. Okur, B. Strockbine, A. Roitberg and C. Simmerling, *Proteins*, 2006, 65, 712-725.
10. P. D. Renfrew, E. J. Choi, R. Bonneau and B. Kuhlman, *PloS one*, 2012, 7, e32637.
11. S. Pronk, S. Pall, R. Schulz, P. Larsson, P. Bjelkmar, R. Apostolov, M. R. Shirts, J. C. Smith, P. M. Kasson, D. van der Spoel, B. Hess and E. Lindahl, *Bioinformatics*, 2013, 29, 845-854.
12. T. Darden, D. York and L. Pedersen, *The Journal of Chemical Physics*, 1993, 98, 10089-10092.
13. W. Humphrey, A. Dalke and K. Schulten, *Journal of molecular graphics*, 1996, 14, 33-38, 27-38.
14. Schrodinger, LLC, unpublished work.
15. L. Maveyraud, R. F. Pratt and J. P. Samama, *Biochemistry*, 1998, 37, 2622-2628.
16. C. Jelsch, L. Mourey, J. M. Masson and J. P. Samama, *Proteins*, 1993, 16, 364-383.
17. A. M. Simm, A. J. Baldwin, K. Busse and D. D. Jones, *FEBS Lett*, 2007, 581, 3904-3908.
